# Electrochemical Determination of Dexamethasone by Graphene Modified Electrode: Experimental and Theoretical Investigations

**DOI:** 10.1038/s41598-019-47420-0

**Published:** 2019-08-13

**Authors:** Somayeh Alimohammadi, Mohammad Ali Kiani, Mohammad Imani, Hashem Rafii-Tabar, Pezhman Sasanpour

**Affiliations:** 1 Department of Medical Physics and Biomedical Engineering, School of Medicine, Shahid Beheshti University of Medical Sciences, Tehran, Iran; 2 Chemistry & Chemical Engineering Research Center of Iran, Tehran, 14335-186 Iran; 3 Department of Novel Drug Delivery Systems, Iran Polymer and Petrochemical Institute, Tehran, Iran

**Keywords:** Biosensors, Diagnostic devices

## Abstract

We report on a combined experimental and theoretical study concerning the electrochemical behavior of the dexamethasone (DEX) on a graphene modified glassy carbon electrode (GCE). A good agreement between experiments and density functional theory (DFT)-based calculations is observed for the DEX reduction. The electrochemical behavior of the DEX was investigated on the surface of a glassy carbon electrode (GCE) modified with different type of graphenes, including graphene quantum dot (GQD), graphene oxide (GO), electrochemically synthesized graphene (EG), graphene synthesized by the Hummer method (HG) and graphene nanoplate (GNP) using voltammetric techniques (CV, DPV and SWV). The results exhibited a significant increase in the reduction of the peak current of the DEX in  the GNP modified GCE (GNP/GCE) in comparison to other modified electrodes and bare GCE. The unique morphology, size and electro catalytic properties of the GNP cause a sensitive response of the DEX in a novel sensor. Under the optimized experimental condition, the GNP/ GCE showed two linear dynamic ranges of 0.1–50 μM and 50–5000 μM with a low detection limit of 15 nM for determination of the DEX. The novel sensor is successfully applied to the sensitive determination of the DEX in human plasma samples with satisfactory recoveries. Energy of the LUMO and HUMO orbitals and energy calculations for the DEX molecule interacting with graphene were performed using the density functional B3LYP/6–31G. The theoretical results allied to significant charge transfer took place due to the interaction of the DEX with the applied graphene.

## Introduction

The Dexamethasone (DEX) is one of the synthetic glucocorticosteroids with a minimum of mineralocorticoid activity. The DEX is known as a potent anti-inflammatory drug^1^. It is often used in a preoperative setting, including prophylaxis against postoperative nausea and vomiting, reduction of the airway and cerebral edema, and it might be useful to moderate acute and chronic pain^2^. Considering the strong efficacy against the diseases, it has been reported that the overdose of the DEX levels in the blood could result in a number of adverse effects including seizures, gastrointestinal perforation, and heart attack. Long-term usage can also cause Cushing’s syndrome, osteoporosis, and cataracts^3,4^. Monitoring the dose inside the body might be essential and an applicable approach in this regard. The applied dose of the DEX is much smaller than other synthetic corticosteroids^5^. Therefore, a sensitive method is required for the determination of the DEX in plasma after administration for the efficient and nontoxic administration of the DEX. To address this issue, numerous studies have been reported using various methods for its determination in a highly sensitive and selective manner. Among the currently available applied methods, i.e., radioimmunoassay, liquid chromatography-tandem mass spectrometry, stable isotope dilution mass spectrometry, and high performance liquid chromatography, chemiluminescence and thin-layer chromatography^6–12^, the electrochemical based techniques, in view of their response time and cost, are regarded as one of the most efficacious tools for detection of the DEX^13^. The DEX is an electroactive compound that could be reduced at specific known electrical potentials, and its electrical property could be used as an indicator for its detection.

There have been limited studies using electrochemical techniques for determination of the DEX^5,14–21^. Initially, Jeyaseelan *et al*. used the differential pulse polarographic method for the trace determination of the dexamethasone sodium phosphate^14^. Oliveira *et al*. developed a square wave adsorptive stripping voltammetry at hanging mercury drop electrode for the determination of the DEX in a Britton–Robinson buffer at pH 2.0^16^. They observed two well-defined reduction peaks in CV’s study and presented a mechanism for the events involved in the reduction process. Appropriate figures of merit (LOD = 2.54 nM and LOQ = 8.47 nM) have been reported in the study for determination of the DEX. Despite all this, this method is not suitable for a quantitative determination of the DEX in a biological sample due to the use of mercury and the high acidity of the environment.

Goyal *et al*. used an edge plane pyrolytic graphite electrode (EPPGE) modified with a fullerene-C_60_ for the determination of the DEX by using the square wave voltammetry. In the modified electrode, the reduction of the peak current of the DEX increased 4 times in comparison with the bare EPPGE^5^. In another study, Rajendra N. Goyal investigated the effect of the cetyltrimethylammonium bromide (CTAB) on the electrochemical determination of the DEX at an EPPGE modified with a single-walled carbon nanotube (SWCNT). The reduction response of the DEX was increased in the modified electrode because of a unique electrocatalytic property of the SWCNT. In general, the investigation of the oxidation and reduction behavior of the DEX is complicated and limited. A major problem in the dexamethasone oxidation pathway is the overlapping peak of the oxidation of other biological species (e.g., uric acid (UA), ascorbic acid (AA), catecholamine molecules). Also for giving the oxidation signal, an acidic media is required so its application is limited in a real sample. As mentioned above, in recent years the modification of carbonic substrates with various carbon nanotubes^15^ and fullerenes^5^ has been reported and the modified electrodes have been used to study the reduction signal of the DEX.

Graphene is the recent member of the multi-dimensional carbon material family. It is the atomically thinnest and lightest two-dimensional carbon nanomaterial composed of sp^2^- carbon bonded atoms. The combination of the extraordinary properties of graphene, such as its high electron mobility, a large surface area (theoretically 2630 m^2^/g for single layer)^22^, the high current density, the high stability and excellent thermal conductivity^23^, make it an ideal platform for fabrication of various modified electrodes for use in electrochemical sensors and biosensors^24^.

In this study, different types of graphene, including graphene quantum dot (GQD), graphene oxide (GO), electrochemically synthesized grapheme (EG), reduced graphene synthesized by the Hummer (HG) method and graphene nanoplate (GNP) were used to modify the glassy carbon electrode for determination of the DEX. The results of the differential pulse voltammetry studies exhibited a significant increase in the reduction of the peak current of the DEX on the surface of the GNP modified electrode compared to the other electrodes. For this reason, the GNP modified electrodes were selected for the voltammetric determination of the DEX in pharmaceutical and clinical samples.

## Results and Discussion

### Morphological properties

**Figure 1:**
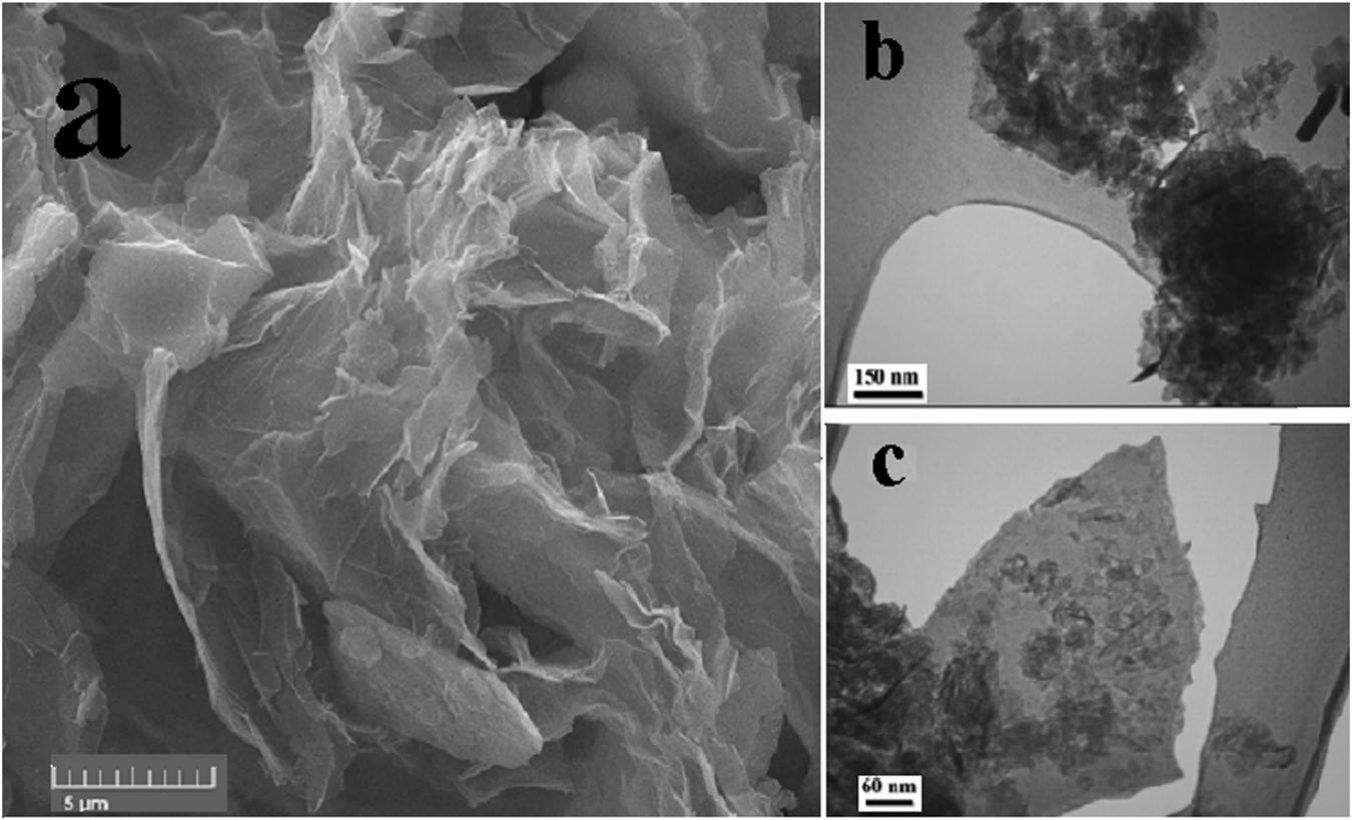
SEM image of electrochemical synthesized graphene (**a**), TEM image of graphene nanoplate (**b**,**c**).

The surface morphology of graphenes was studied by SEM and TEM images. Figure 1a shows the surface morphology of the electrochemically synthesized graphene. The SEM image of the CNT, carbon nanoparticle (CNP), HG and GO are shown elsewhere^25^. As seen in Fig. 1a, the surface of the film exhibits a wrinkled texture associated with the presence of flexible and large lateral size of graphene sheets. Surface morphology of the GNP shown in the TEM images (Fig. 1b,c). Observation under higher magnification revealed the ultra-thin film of the graphene with a small lateral size. The size of the GNP is in the range of 100–400 nm.

### Electrochemical behavior of dexamethasone

**Figure 2:**
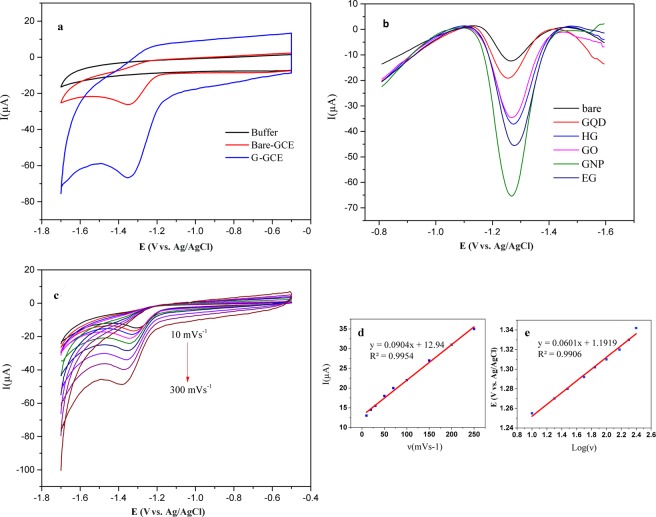
Electrochemical behavior of the DEX. (**a**) A comparison of cyclic voltammograms of 1 mM DEX on the GNP modified GCE, bare GCE and the PBS buffer (pH 7.3) at GCE, Scan rate 100 mVs^−1^. (**b**) The DPV voltammograms of 1 mM DEX on the surface of the GCE modified with different type of graphene in the PBS buffer (pH 7.3) at GCE, scan rate 100 mVs^−1^. (**c**) Cyclic voltammetric curves obtained for 1 mM DEX at the GNP/GCE in the PBS at different scan rates. (**d**) Dependence of peak currents to scan rates. (**e**) Dependence of the peak potential on the log of scan rates.

Initially, the voltammetric behavior of 1 mM DEX in 1 M phosphate buffered saline (pH 7.3) was studied at bare and graphene modified GCE by cyclic voltammetry (CV). The reduction of the DEX at the GCE shows a weak signal at −1.33 V vs. Ag/AgCl. The cyclic voltammogram at the graphene modified GCE exhibits the reduction in the peak at −1.29 V. As shown in (Fig. 2a) the peak of the DEX at the graphene modified GCE is obtained at a less negative potential and the peak current is also enhanced compared with the case of the bare electrode. This significant enhancement of the peak current together with the shift of the peak to less negative potentials obviously demonstrate that the graphene acts as an efficient electron mediator in the electrocatalytic reduction of the DEX, and hence it can be a suitable modifier to enhance the sensitivity in determination of the DEX. As the square wave voltammetry (SWV) and the differential pulse voltammetry (DPV) are known to be more sensitive techniques in comparison to the cyclic voltammetry, they are used in further studies.

### Electrochemical reduction of the DEX on the surface of the various graphene modified electrodes

Electrochemical reduction of 1 mM DEX was investigated on the surface of the GCE modified with different types of graphene by the DPV method (Fig. 2b).

As shown in Table S1, the peak current for all types of graphene-coated electrodes was better than for a bare glassy carbon electrode. Also, the highest peak current of the DEX is obtained on the GNP modified electrode (6 times better than for the bare electrode). This is a proven fact that structural defects and edges increase the catalytic activity. The highest enhancement by GNP is related to the unique size, morphology and structure of the GNP. The GNP has more edges than other forms of graphene (GO, EG, HG and GQD) and the edges have more activity than the plate. Therefore it can be expected to provide the highest current for reduction of the DEX. Also, based on their pure graphitic composition, graphene nanoplatelets are exceptional electrical and thermal conductors. The main reason for this fact is that the multiple or continuous surface electronic states of the GNP will increase the electron-transfer rate for the reduction of the DEX. Therefore, we have used the GNP/GCE as the best-modified electrode for the determination of the DEX in this study.

### Effect of scan rate and frequency

In order to find the mechanism of the electrochemical process, the investigations of scan rate on the peak current were considered. The peak current was found to increase with the scan rate ranging from 10 to 250 mV⋅s^−1^ (Fig. 2c). A linear relation between the current and the scan rates (υ) obtained by Eq. 1 (Fig. 2d) indicated that the reaction of the DEX on the G/GCE endured the adsorption-controlled process. The plot of E versus logν was linear too (Eq. 2) and showed that the electrochemical nature of the reaction was irreversible (Fig. 2e)

1 $${{\rm{I}}}_{{\rm{p}}}({\rm{\mu }}{\rm{A}})=0.090\,{\rm{\upsilon }}\,({\rm{mV}}{{\rm{.s}}}^{-1})+12.94\,({{\rm{R}}}^{2}=0.995)$$

2 $${{\rm{E}}}_{{\rm{p}}}({\rm{mV}})=0.06\,\mathrm{log}\,{\rm{\upsilon }}\,({\rm{mV}}\cdot {{\rm{s}}}^{-1})+1.191\,({{\rm{R}}}^{2}=0.990)$$

The effects of the square wave frequency on the reduction of the DEX and its influence on the peak current and peak potential at the GR-GCE were investigated in the presence of 1 m MDEX in the frequency range of 20 to 300 Hz (Fig. S1a). The results showed that the peak current increased linearly with an increase in the frequency according to Eq. 3 (Fig. S1b). The peak potential was also found to shift to more negative values with increasing frequency. The variation of E with log f can be expressed by the Eq. 4. The plot of E versus logf was linear with a correlation coefficient of 0.998 (Fig. S3c). These results are in agreement with the properties of adsorption controlled irreversible electrochemical process^26^ which also supported the results of the CV.

3 $${{\rm{I}}}_{{\rm{p}}}({\rm{\mu }}{\rm{A}})=0.\,2512\,{\rm{f}}\,({\rm{Hz}})+9.682\,({{\rm{R}}}^{2}=0.997)$$

4 $${{\rm{E}}}_{{\rm{p}}}({\rm{V}})=0.0472\,\mathrm{log}\,{\rm{f}}({\rm{Hz}})+1.194\,({{\rm{R}}}^{2}=0.998)$$

### Quantum-chemical studies

**Figure 3:**
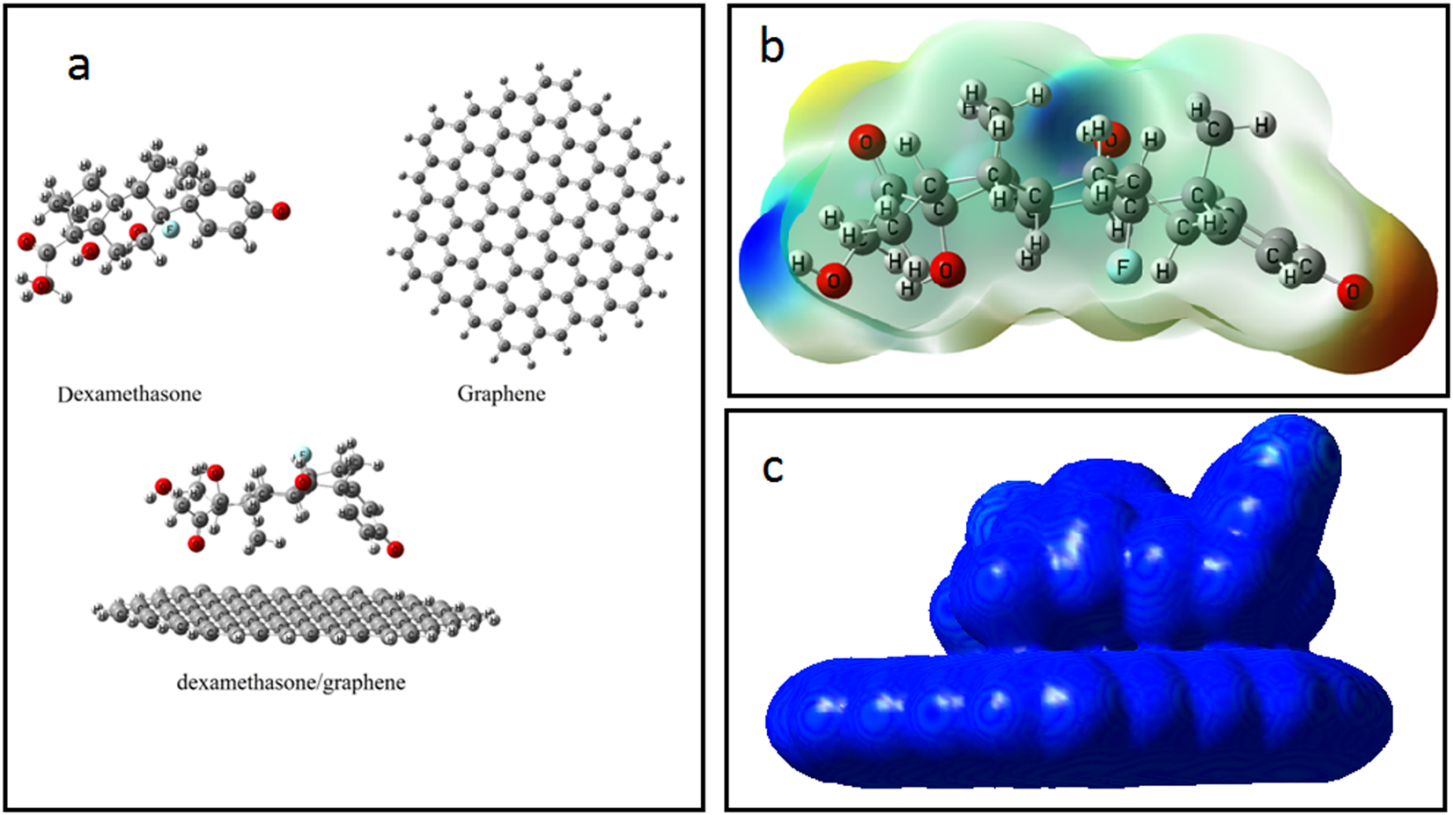
(**a**) Structures of optimized DEX, graphene and DEX/graphene. (**b**) The MEP plot of the DEX. (**c**) Computed total electron density plots for adsorption properties of DEX on the graphene.

**Figure 4:**
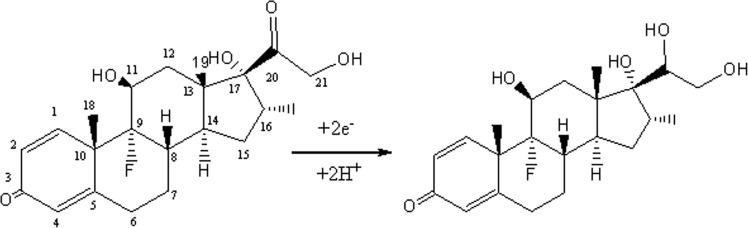
Proposed suggested for the reduction of the DEX.

Figure 3a shows the optimized structure of the DEX, the graphene and the DEX/graphene using the B3LYP computational method. The adsorption energy of the DEX on the graphene was calculated to be −7.51 kcal mol^−1^ (Table 1). After the adsorption process, the dipole moment of the complex is increased from 0.00 in the pristine graphene to 4.14 Debye, showing that the polarity of the complex is increased. The HOMO and LUMO values are reduced from−4.65 and −2.53 eV in the graphene to −4.56 and −2.46 eV in complex condition. The HOMO represents the ability to donate an electron and LUMO as an electron acceptor. The relevant values of the LUMO–HOMO energy gap are given in Table 1, indicating that the adsorption of the dexamethasone/graphene complex has no significant effect on the electronic properties of the graphene. Figure 3b illustrates the molecular electrostatic potential (MEP) plot for the DEX molecule. As shown, the partial negative charges are mainly localized over the carbonyl group (red color), which can interact with the graphene. Total electron density plots for the most stable configuration are displayed in Fig. 3c. As can be easily seen from Fig. 3c, a significant charge transfer has occurred due to the interaction of the DEX molecule with the graphene. Therefore, from the quantum-chemical point of view, there is a tow site for reduction processes. These results are in accord with the previous experimental study, which pointed out that the ketone groups at positions C-3 and C-20 are feasible sites for the reduction of the DEX^16,21^. It has been previously reported that ketosteroids with a carbonyl group conjugated with a double bond, undergoe a reduction in comparison with the isolated carbonyl group. The reduction of unconjugated carbonyl group has also been reported to be activated by the neighboring hydroxyl groups^27^. The variation in UV absorption through controlled potential electrolysis of a similar compound^28^ supported the observation that the keto group conjugated with the double bond remains intact and the reduction in the DEX takes place at position 20 as shown in Fig. 4. The carbonyl group at position 20 is activated by the hydroxyl groups present at positions 17 and 21.

**Table 1:** Calculated adsorption energy (E_ad_), HOMO energies (EHOMO), LUMO energies (ELUMO), energy gap (E_g_) and dipole moment (DM) of systems.

NAME	E_ad_ (kcal mol^−1^)	E_HOMO_ (eV)	E_LUMO_ (eV)	E_g_ (eV)	DM (Debye)
Dexamethasone	—	−6.15	−1.37	4.77	7.22
Graphene	—	−4.65	−2.53	2.11	0.00
dexamethasone/graphene	−7.51	−4.74	−2.63	2.10	4.14

HOMO: highest program package occupied molecular orbital, LUMO: lowest unoccupied molecular orbital, E_ad_: adsorption energy, DM: dipole moment.

### Effect of Buffer composition

**Figure 5:**
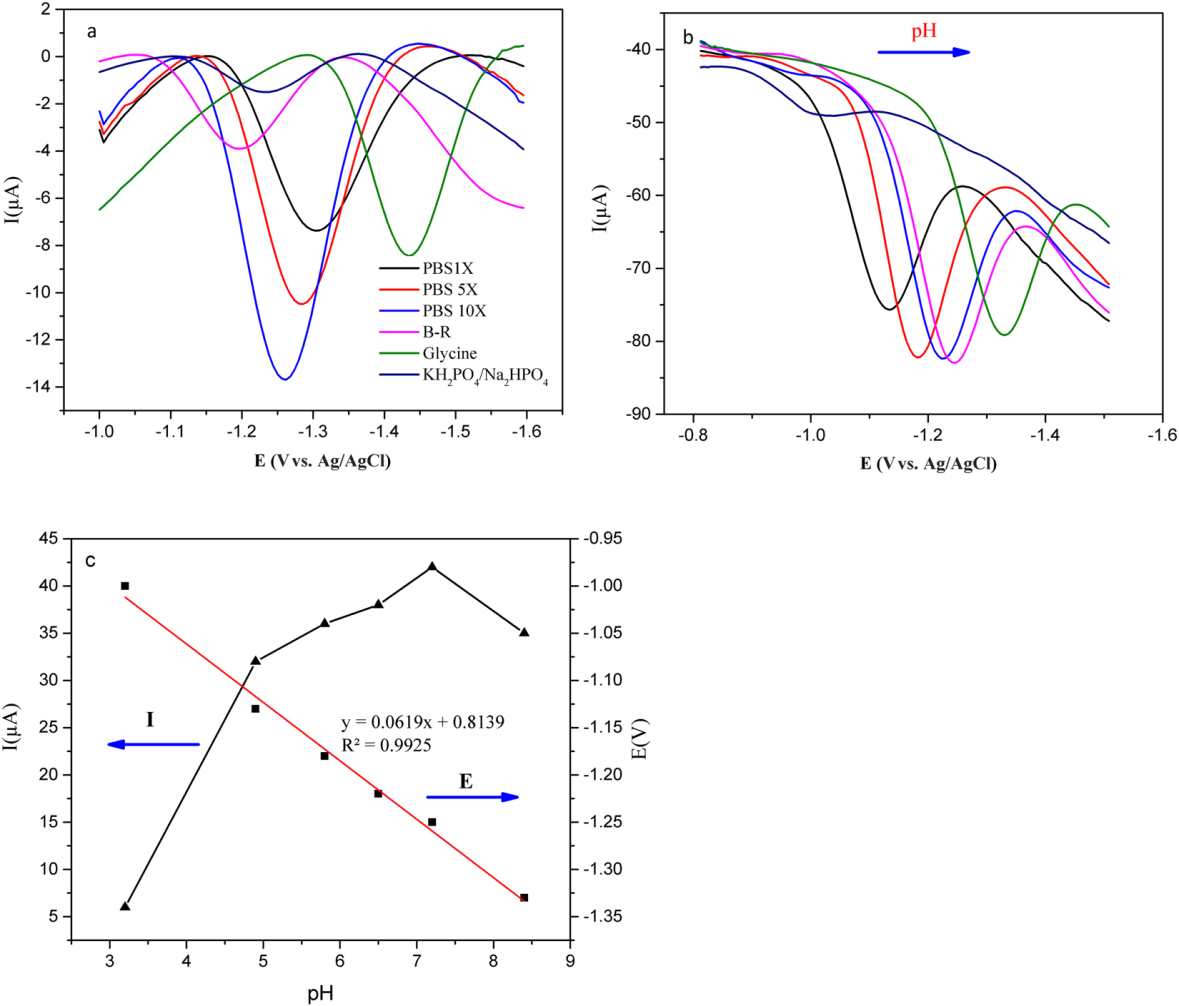
Electrolyte effect. (**a**) A comparison of the DPV voltammograms of 0.1 mM DEX on the GNP modified GCE in different electrolytes in same pH (7.3). (**b**) DPV voltammograms for 0.5 mM DEX on GNP/GCE in the PBS solutions at different pH: 3.0–8.5 at a scan rate of 100 mV s^−1^ (**c**) Plot of pH versus peak current and Plot of Ep versus pH value.

The composition of the electrolyte is one of the important factors in the electrochemical behavior of compounds. We have used the Britton-Robinson buffer, the phosphate buffer, the glycine buffer and the phosphate saline buffer as electrolyte for determination of the DEX (1 mM) on the surface of the GNP/GCE electrode in the same pH (7.3) by the DPV. As can be seen in Fig. 5a, the current peak and the reduction potential of the DEX are dependent on the type and the concentration of the buffer. Among different buffer solutions, it is shown that the reduction in the current peak has its maximum in the PBS (10X). This result shows that the electron transfers between the GNP/GCE and the DEX occur in the high capacity buffer better than the low capacity buffer. Therefore, the PBS (10X) was selected as the buffer solution for determination of the DEX.

The pH of the electrolyte has a significant influence on the response signal of the DEX. The results of our study show that, the peak of the DEX moved to the less negative potential by decreasing the pH of the electrolyte. Simultaneously, the peak current reached the maximum value when the pH was set to 7.3 (Fig. 5b). The potential (E) was shifted linearly with the electrolyte pH in the range of 3 to 8.5 (Fig. 5c). Linear regression of the DEX can be expressed by Eq. 5:

5 $${{\rm{E}}}_{{\rm{p}}}({\rm{mV}})=61\,{\rm{pH}}+813\,({{\rm{R}}}^{2}=0.992)$$

The slope of E versus pH is 61 mV/pH. This value is close to the theoretical value of 59 mV/pH which suggested that the number of proton transfer is equal to the number of electrons in the DEX reaction.

### Optimization of the DPV parameters

The pulse amplitude, the interval time (time elapsed between two following pulses), the pulse time (time elapsed between the pulse application and the current measurement) and the step potential (potential increase between two successive current measurements) have been considered as important parameters to be optimized for sensitive determination of the DEX by the DPV method. The parameter values have been chosen in order to significantly adjust the peak current, peak width and peak of potential.

As shown in Fig. S2 the peak current increases with an increase in the pulse amplitude in the range of 20–200 mV with the Ip value of 5–140 μA for 1 mM of the DEX. The variation in the peak of the potential is negligible but the peak width increased with increasing the pulse amplitude. Since the large peak width is not suitable for the analysis of the low amount of the DEX, 100 mV was chosen for the pulse amplitude. The effects of the interval time were investigated in the range of 100–500 ms (Fig. S3). The peak current decreases by increasing the interval time without any change in the peak of potential and negligible change in the peak width. As a result, 100 ms is the most appropriate choice for the interval time. The peak current decreased with the increase in the pulse time in the range of 2–15 ms without any change in the peak of the potential and the peak width (Fig. S4). As shown in Fig. S5, the effect of the step potential in the range of 2–20 mV was studied. According to the results, the peak of the current is increased with increasing the step potential. In this regard, the pulse time of 2 mS and the pulse amplitude of 14 mV were selected for further studies, since the highest peak current was observed at these values.

### Analytical performance of the sensor

**Figure 6:**
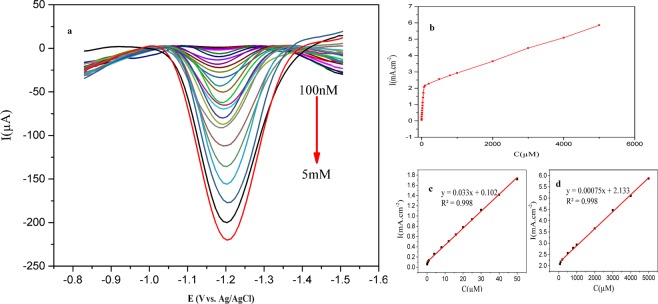
Calibration analysis. (**a**) DPV voltammogram obtained from the GNP/GCE in the presence of the DEX ranging from 100 nM to 5 mM in pH 7.3 PBS. (**b–d**) calibration plots of the peak current vs. different concentrations of the DEX.

In order to evaluate the proposed sensor for quantitative analysis of the DEX, under the optimal conditions, the DPVs were obtained for measurements of continuous injection of the DEX into the PBS buffer. The peak current increases with increasing the DEX concentration in 100 nM–5 mM at two intervals (Fig. 6).

Eqs. 6 and 7 show the linear regression:

6 $$100\,{\rm{nM}}\,-\,50\,{\rm{\mu }}{\rm{M}}:\,{{\rm{I}}}_{{\rm{p}}}({\rm{mA}}/{{\rm{cm}}}^{2})=0.033\,{\rm{C}}\,({\rm{\mu }}{\rm{M}})+0.102\,({{\rm{R}}}^{2}=0.998)$$

7 $$50\,{\rm{\mu }}{\rm{M}}\,-\,5\,{\rm{mM}}:\,{{\rm{I}}}_{{\rm{p}}}({\rm{mA}}/{{\rm{cm}}}^{2})=0.00075{\rm{C}}\,({\rm{\mu }}{\rm{M}})+2.133\,({{\rm{R}}}^{2}=0.998)$$

Based on this, the sensitivity of the proposed method was found to be 33 (μA μM^−1^ cm^−2^). The limit of detection was calculated by using the formula 3s/σ where s is the relative standard deviation (RSD) of a blank buffer (for five) and σ is the slope of the calibration curve and was found to be 1.5 × 10^−8^ M. Compared with the previous reports in Table 2, the proposed DEX sensor exhibits a low limit of detection (LOD) as well as a high sensitivity and a broad range of detection. The outstanding performance of sensing might be due to the unique size and morphology and purity of the GNP.

**Table 2:** Comparison of analytical parameter of electrochemical sensors for the DEX determination.

Electrode	Technique	Linear range (M)	Detection limit (M)	Potential (V)	Refs
C60/EPPGE	SWV	5 × 10^−8^ −1 × 10^−4^	5 × 10^−8^	−1.25	^5^
HMDE	DPV	8.5 × 10^−5^ −1.4 × 10^−5^	7.6 × 10^−6^	−1.14	^14^
SWCNT/CTAB/EPPGE	SWV	1 × 10^−9^ −1 × 10^−4^	1 × 10^−9^	−1.2	^15^
HMDE	SWV	4.9 × 10^−8^ −6.1 × 10^−7^	2.5 × 10^−9^	−0.85, −0.2	^16^
MWCNT/PE	SWV	0.15 × 10^−8^ −1 × 10^−4^	0.09 × 10^−6^	+0.8	^17^
GCE/GNP	DPV	1 × 10^−7^ −5 × 10^−3^	1.5 × 10^−8^	−1.3	This work

DPV: differential pulse voltammetry, SWV: square wave voltammetry, EPPGE: edge plane pyrolytic graphite electrode, HMDE: hanging mercury electrode, SWNT: single-walled carbon nanotubes, CTAB: cetyltrimethylammonium bromide, MWCNT: Multiwall carbon nanotube, PE: pencil electrode, GCE: glassy carbon electrode, GNP: graphene nanoplate.

### Selectivity and stability

The selectivity analysis reveals that, due to the electrolyte composition and concentrations which are used in this study, the proposed sensor was free from the interference of common ions (Na^+^, Cl^−^, K^+^, NO3^−^, PO4^3−^, NH^4+^, Mg^2+^ and Ca^2+^). The reduction potential (−1.29 V) of the DEX in the proposed structure, makes the sensor show selectiveness in biological samples over the sucrose, glucose, ascorbic acid, uric acid, dopamine, else. The only natural substance in the body with a similar structure to the DEX is hydrocortisone. As shown in Figs S6 and S7 the reduction potential for hydrocortisone is distinguished from the DEX, also the different amount of hydrocortisone has no effect on the current peak of the DEX (Fig. S8).

In order to evaluate the stability of the sensor, the GNP modified GCE electrode was operated for several times in 60 days for detection of 0.5 mM DEX and the RSD was 0.4%. After storing for 60 days in ambient condition, the peak current maintained 97.8% of its initial value (Fig. S9). All measurements revealed the proper stability and recovery of the GNP/GCE sensor.

### Real sample analysis

In order to verify the reliability of the fabricated sensor for the determination of the DEX in clinical samples, the electrode performance was investigated in human blood serum without any further treatment (analyte addition approach). In this regard, the standard addition was carried out by spiking a specific amount of the DEX standard solution into sample. The DPV signal apparently increased after being spiked by standard solutions containing different concentrations of the DEX. The calibration plot of the peak current versus the DEX concentration is shown in Fig. S8a. Two linear regions are seen again (Fig. S10b,c) and Eqs. 8 and 9 show the linear regression.

8 $$200\,{\rm{nM}}\,-\,50\,{\rm{\mu }}{\rm{M}}:\,{{\rm{I}}}_{{\rm{p}}}({\rm{mA}}/{{\rm{cm}}}^{2})=0.0288{\rm{C}}\,({\rm{\mu }}{\rm{M}})+\,59.11\,({{\rm{R}}}^{2}=0.998)$$

9 $$50\,{\rm{\mu }}{\rm{M}}\,-\,2\,{\rm{mM}}:\,{{\rm{I}}}_{{\rm{p}}}({\rm{mA}}/{{\rm{cm}}}^{2})=0.00082\,{\rm{C}}\,({\rm{\mu }}{\rm{M}})+1550\,({{\rm{R}}}^{2}=0.994)$$

Based on this, the limit of detection and the sensitivity of the proposed sensor were found to be 59 nM and 28 (μA μM^−1^ cm^−2^) in human plasma samples respectively. It can be observed that the fabricated sensor exhibits an excellent broad linear range. These results may be due to the unique property of the GNP such as the high surface-to-volume ratio with more electroactive sites of the GNP for the DEX molecules, although the sensitive catalytic performance is due to the promoted electron transfer and considerable catalytic activity.

## Conclusion

In this study, different electrode structures were fabricated by modifying the surface of the GCE with various graphenes, including GQD, GO, EG, HG and GNP and their performance in detection of the DEX were investigated. Among various types, the GNP/GCE showed a better electrochemical response for the DEX. It could be due to the unique size, morphology and purity of the GNP which cause the high current density, high stability and excellent electrical conductivity of the GNPs. In order to have the best results, most of the electrochemical parameters were optimized. In addition, theoretical analyses were used for better understanding of the mechanistic electrochemical aspects of the DEX and the graphene. All results were in full agreement with the properties of adsorption controlled irreversible electrochemical process. Under the optimized condition for voltammetric determination of the DEX, two linear dynamic ranges of 100 nM–50 μM and 50 μM–5 mM with high sensitivity could be determined. Comparing with the recent studies on the DEX sensors, the proposed structure of the sensor showed a quite lower detection limit of 15 nM as well as a wider linear range of 100 nM–5 mM. It exhibited a good selectivity, sensitivity and high stability. The proposed sensor was applied for the detection of the DEX in human plasma samples without any treatment with high sensitivity and acceptable recovery results. As the analysis is based on reduction, the common interfering compounds in human plasma did not interfere in the detection. Based on our findings, the novel proposed sensor could be used for continues determination of the DEX in pharmaceutical and biological samples with satisfactory results.

## Methods

### Reagents

The DEX and hydrocortisone were obtained from Alborzbalk pharmaceutical company (Tehran, Iran) and were used as received without further purification. N, N-dimethylformamide (DMF) was purchased from the Merck. GNP (XG sciences) and CNPs (ca. 9–18 nm diameter, Emperor 2000, Cabot Corporation) were obtained commercially. Phosphate saline buffers of appropriate pH and ionic strength were prepared from analytical grade of NaH_2_PO_4_, Na_2_HPO_4_, NaCl and KCl salts (Merck) according to the method of Christian and Purdy^29^. Natural graphite powder (325 mesh) was purchased from the Merck. All aqueous solutions were prepared with deionized water. A stock solution of the DEX (10 mM) was prepared in buffer and kept in the dark. Required concentrations of the DEX in aqueous buffer solutions were then prepared from the stock solution diluted by appropriate buffer solutions. A fresh solution of the DEX was prepared just prior to use. Graphene was synthesized by modified Hummers method^30^ and electrochemical synthesis method^31^.

### Apparatus

Electrochemical experiments were carried out using an AUTOLAB PGSTAT100 (Eco Chemie, Netherlands). The system was run by a PC via the GPES 4.9 software. A conventional three-electrode glass cell was used with a platinum wire as an auxiliary electrode, an Ag/AgCl (3MKCl) electrode as a reference electrode and the working electrode was bare or modified GCE (∼2 mm). All potentials reported are referred to the Ag/AgCl electrode at an ambient temperature. The pH of the buffers was measured using a Metrohm744. Digital pH-meter. TESCAN Vega3 Model scanning electron microscopy (SEM) and Zeiss-EM10C–100 KV transmission electron microscopy (TEM) instruments were used for analysis of the surface morphology of the graphene.

### Preparation of the modified glassy carbon electrodes

Prior to the modification of the glassy carbon electrode, its surface was polished with 0.05 µm alumina (BAS) using micro cloth pad until a mirror-like surface was obtained. Then, it was sequentially sonicated in double-distilled water and ethanol for 5 minutes and washed several times with double distilled water. After this, the electrodes were ready for modification.

The GNP, EG, GQD, HG and GO suspensions were prepared by dispersing 1 mg of the preferred carbon nanomaterial in 1 mL of the DMF solvent under ultrasonic agitation for 2 hours. Then, 10 µL of each suspension was taken with a micropipette and was cast on the electrode surface, the electrode was placed under the IR lump for 30 minutes to evaporate the DMF solvent.

For the electrochemical preparation of the RGO modified electrode, the GCE as the working electrode was immersed in 10 ml of LiClO_4_ (0.1 M) containing 3.0 mg/ml GO, and then subjected to a constant potential of −1.2 V for 300 seconds. After deposition, the graphene modified GCE was dipped into the ultrapure water to remove the residual GO and LiClO_4_. Finally, the GCE was dried under the IR lump to produce the compact graphene layer^27–33^. Before the voltammetric measurements, the modified electrode was cycled 15 times between + 1.6 to –1.6 V (scan rate of 100 mV.s^−1^) in a phosphate saline buffer solution (pH 7.3) to renew the electrode surface.

### Computational method

Theoretical and experimental studies have shown the importance of quantum-chemical studies to understand the mechanistic electrochemical aspects of various compounds. Here, this information was used to determine the probable sites of the reduction of the DEX and to investigate the interaction between the graphene and the DEX. All calculations reported in this study were obtained by applying the density function theory (DFT) method. Also all calculations of optimization, electrical, and energy calculations for dexamethasone molecule interacting with the graphene were performed using the B3LYP/6-31G* level of theory^34,35^ with the Gaussian03 package^36^. Initially, the calculations of the HOMO (highest occupied molecular orbital) and LUMO (lowest unoccupied molecular orbital) values of the DEX and the graphene before and after the adsorption reaction were performed. Next, Mülliken’s charges for finding the probable atoms responsible for the reduction processes were calculated.

The adsorption energy (E_ad_) of the DEX molecule on the graphene is computed using Eq. 10:

10 $${{\rm{E}}}_{{\rm{ad}}}={{\rm{E}}}_{{\rm{DEX}}/{\rm{Graphene}}}-[{{\rm{E}}}_{{\rm{Graphene}}}+{{\rm{E}}}_{{\rm{DEX}}}]$$

where E_DEX/Graphene_ is the total energy of the graphene interacting with the DEX molecule, E_Graphene_ and E_DEX_ are the total energies of the graphene and dexamethasone, respectively.

## Supplementary information

Supplementary Material.
