# Supplementary material for: Electrochemical Determination of Dexamethasone by Graphene Modified Electrode: Experimental and Theoretical Investigations
Source: Sci Rep. 2019 Aug 13;9:11775. doi: 10.1038/s41598-019-47420-0 (PMC6692413; doi:10.1038/s41598-019-47420-0)
Supplement: Supplementary file 1 — Supplementary Material. [file 41598_2019_47420_MOESM1_ESM.docx]

## Electrochemical Determination of Dexamethasone by GrapheneModified Electrode: Experimental and Theoretical Investigations

Somayeh Alimohammadi^1^, Mohammad Ali Kiani^2*^, Mohammad Imani^3^, Hashem Rafii-Tabar^1^, Pezhman Sasanpour^1*^

1- Department of Medical Physics and Biomedical Engineering, School of Medicine, ShahidBeheshti University of Medical Sciences

2- Chemistry & Chemical Engineering Research Center of Iran, Tehran 14335-186, Iran

3- Department of Novel Drug Delivery Systems, Iran Polymer and Petrochemical Institute, Tehran, Iran

*Corresponding Authors: [makiani@ccaerci.ac.ir](mailto:makiani@ccaerci.ac.ir),[pesasanpour@sbmu.ac.ir](mailto:pesasanpour@sbmu.ac.ir)

**Supplementary Materials**

**Table S1.** Comparison of peak current of the DEX reduction on the surface of the GCE modified with the various type of graphene by the DPV method

| Modifier | Bare | GQD | GO | HG | EG | GNP |
| --- | --- | --- | --- | --- | --- | --- |
| Current(µA) | 12 | 19 | 32 | 38 | 46 | 65 |

GQD: graphene quantum dot, GO: graphene oxide, EG: electrochemical synthesized

grapheme, HG: reduced graphene synthesized by hummer method and GNP: graphenenanoplate.


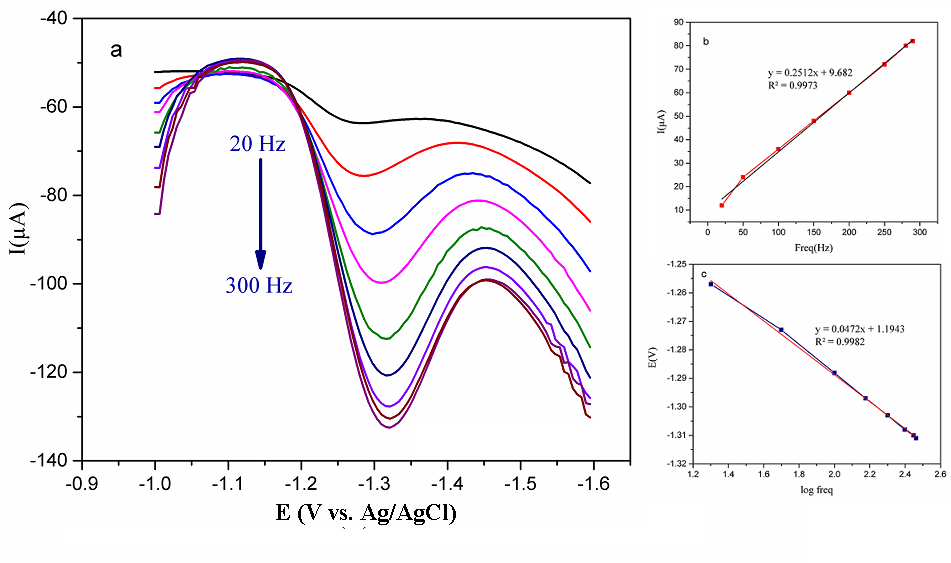


Fig. S1 Square wave voltammograms of 1 mM DEX at the GNP/GCE in the PBS at different frequencies in pH 7.3 (a). Dependence of the peak currents on frequency (b). Dependence of peak potential to log of frequency (c).


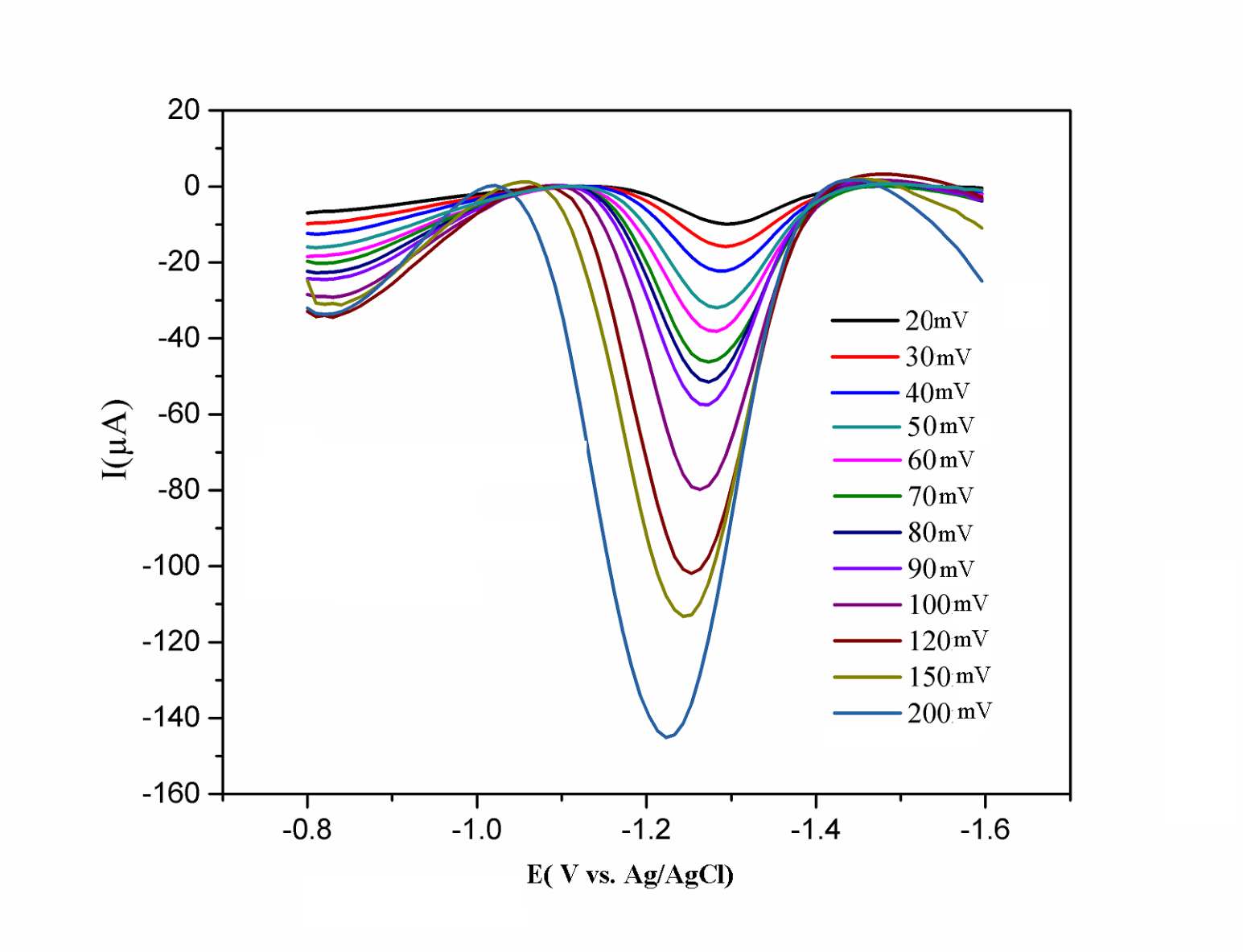


Fig. S2 DPV voltammograms for 0.5 mM DEX on GNP/GCE in the PBS solutions (pH 7.3) at different pulse amplitude.


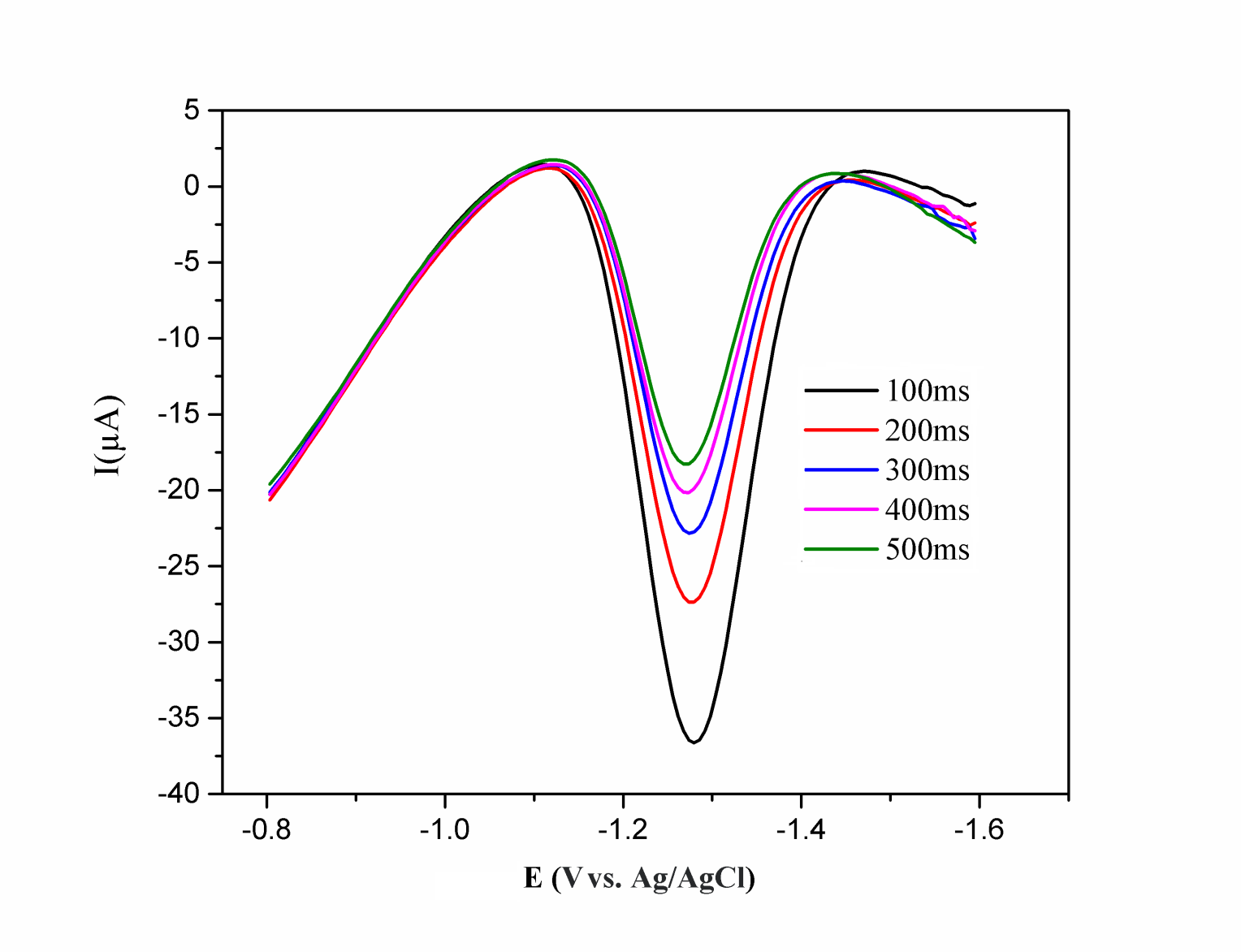


Fig. S3 DPV voltammograms for 0.5 mM DEX on the GNP/GCE in the PBS solutions (pH 7.3) at different interval time


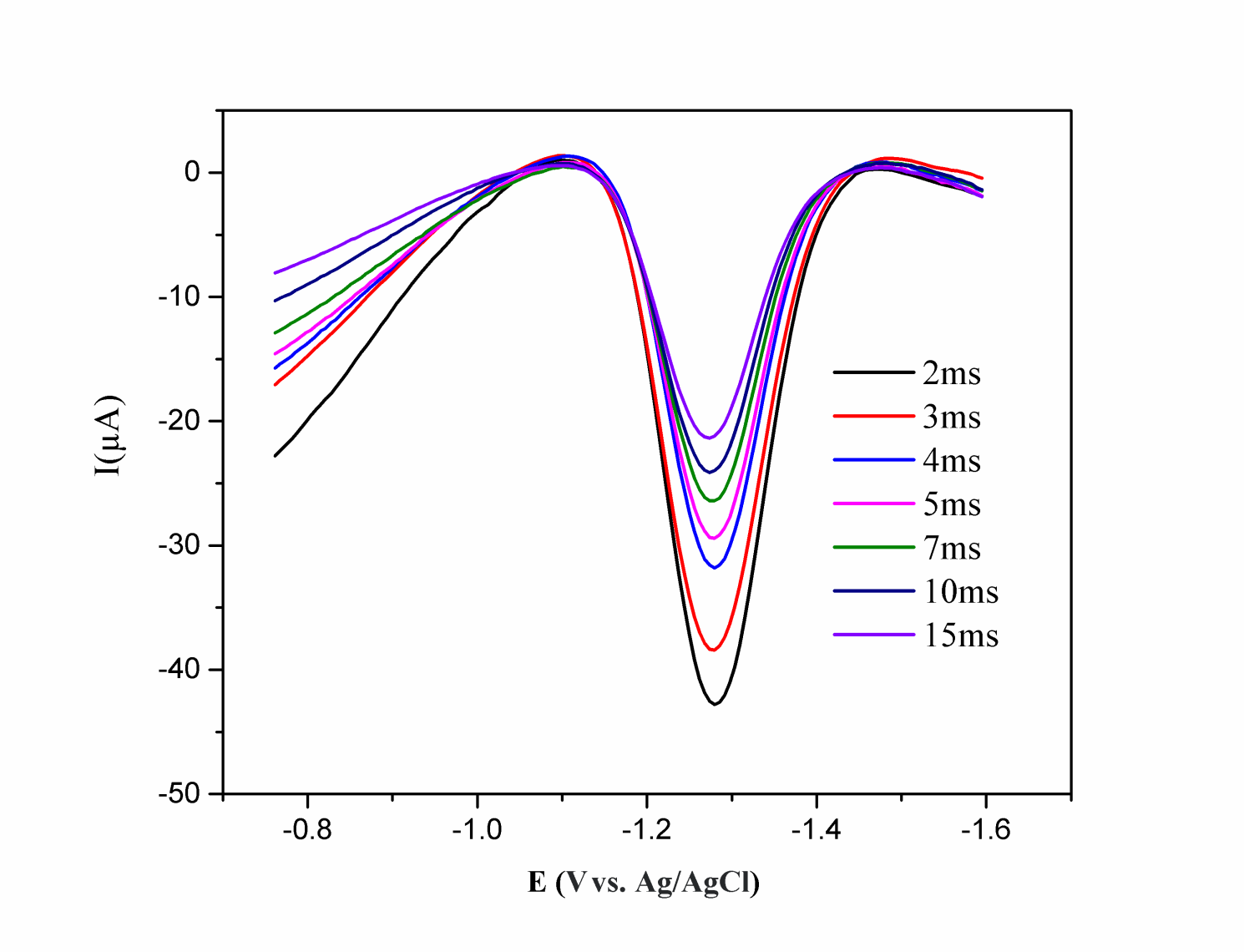


Fig. S4 DPV voltammograms for 0.5 mM DEX on the GNP/GCE in the PBS solutions (pH 7.3) at different pulse time


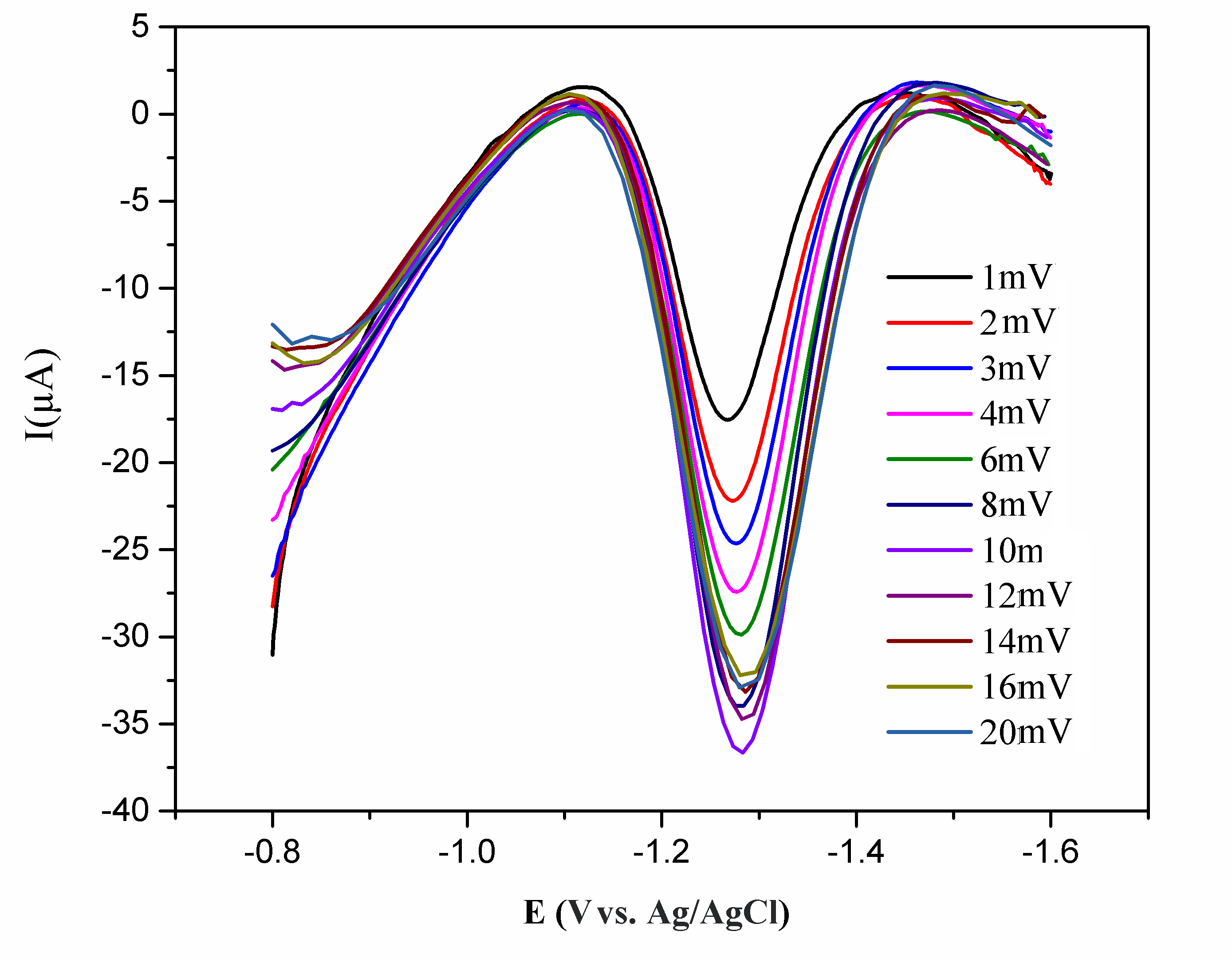


Fig. S5 DPV voltammograms for 0.5 mM DEX on the GNP/GCE in the PBS solutions (pH 7.3) at different step potential


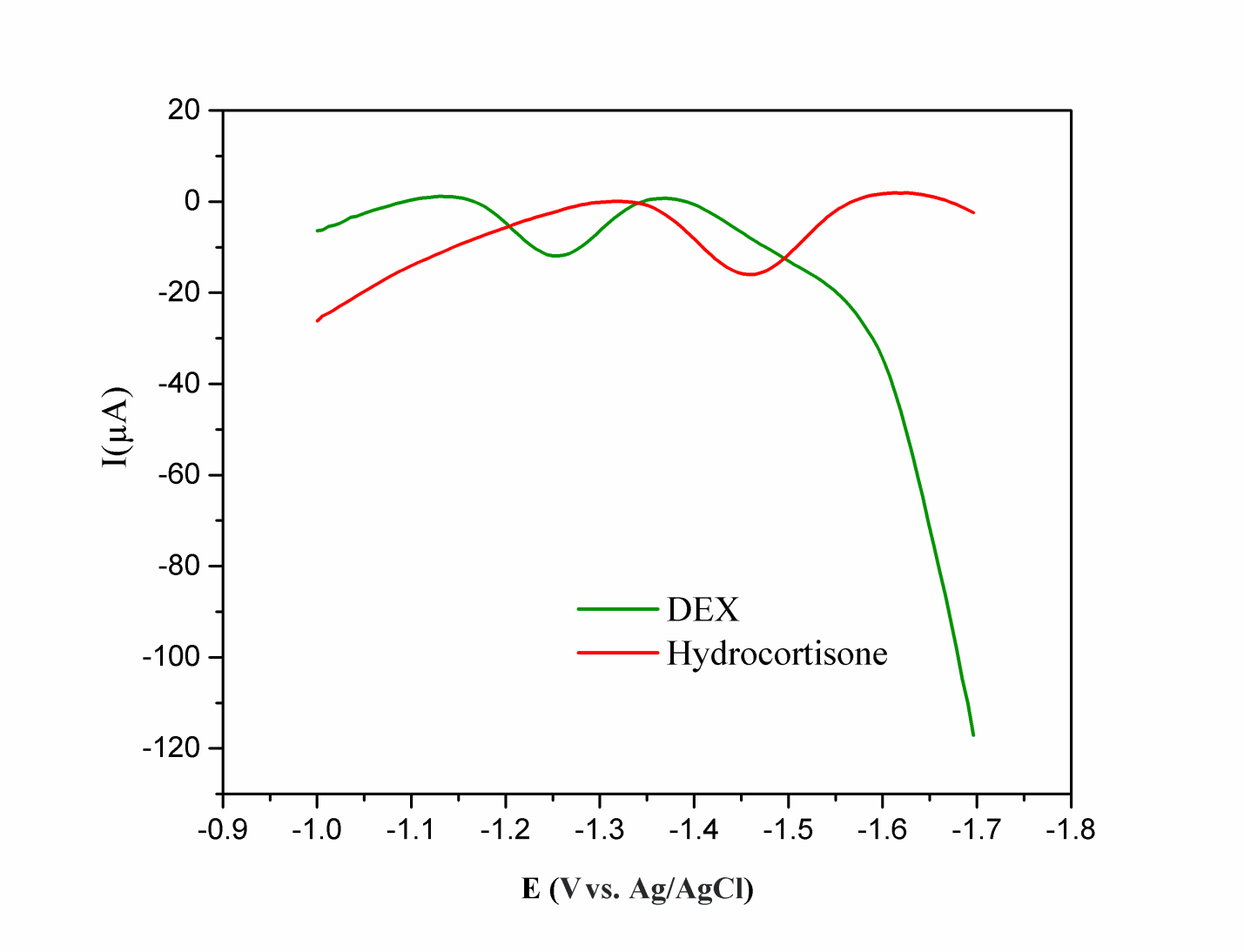


Fig. S6 DPV voltammogram obtained from GNP/GCE in the 1mM DEX and 1mM hydrocortison in the PBS solutions (pH 7.3).


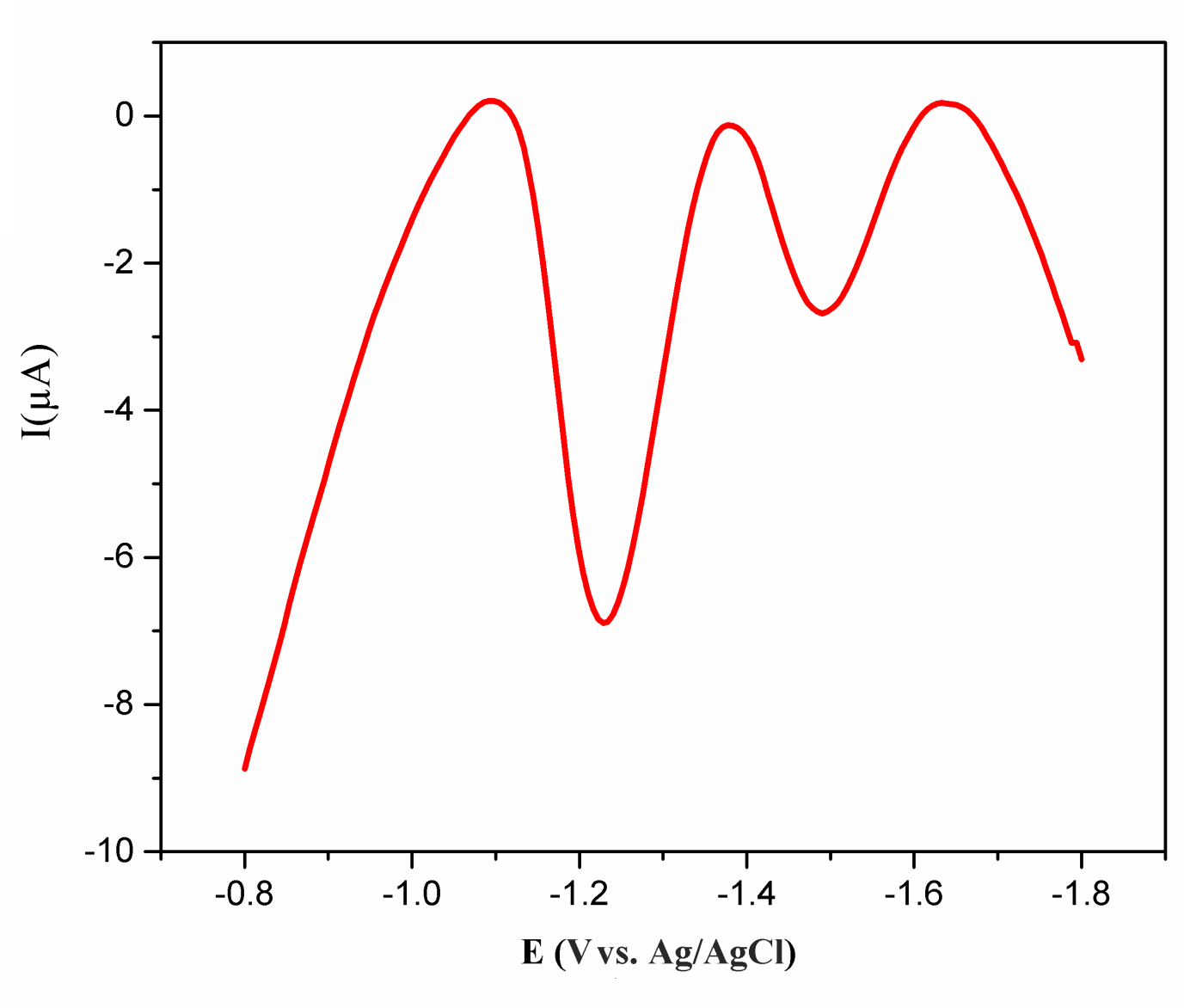


Fig. S7 DPV voltammogram obtained from GNP/GCE in the presence of 0.1mM DEX and 0.1mM hydrocortison in the PBS solutions (pH 7.3).


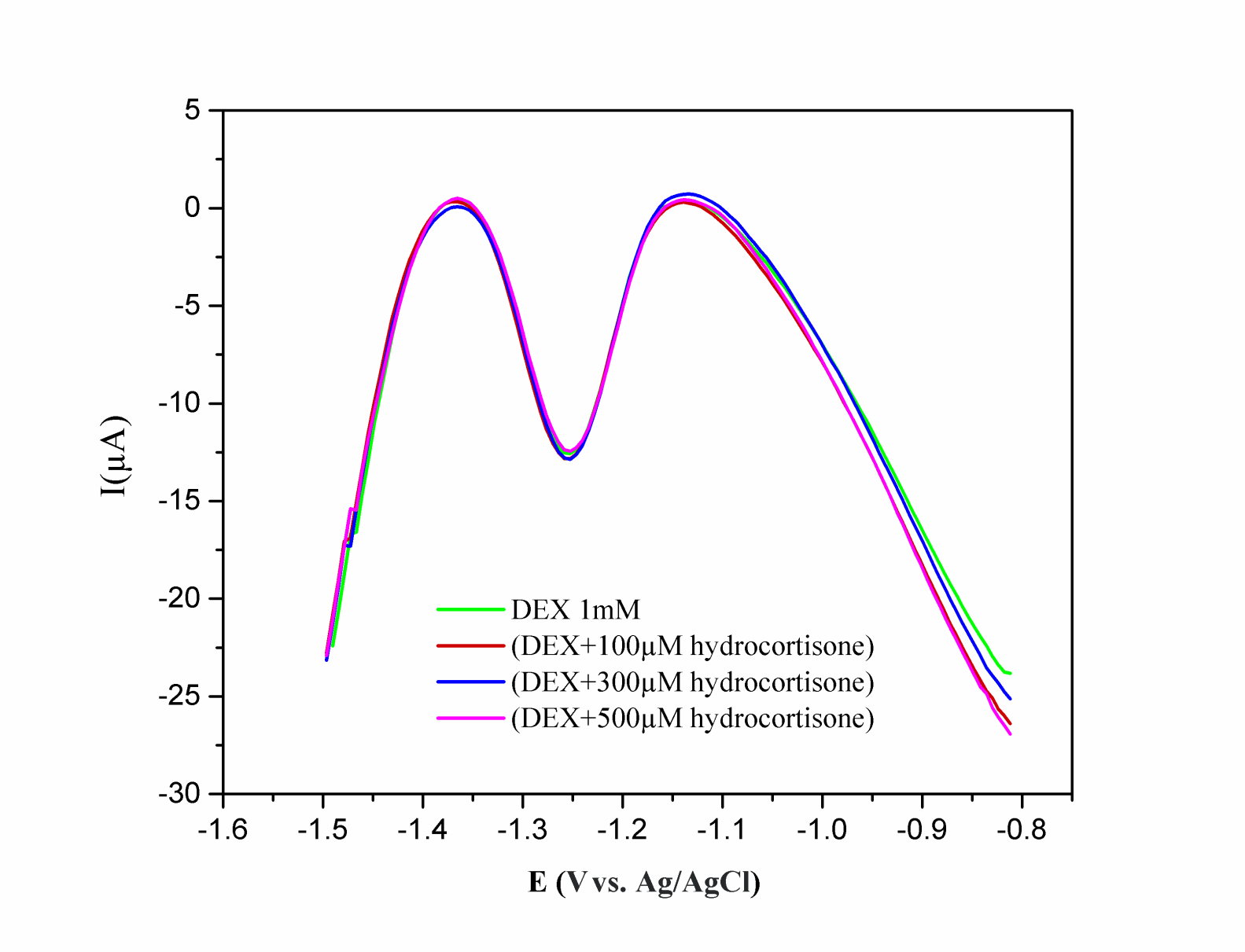


Fig. S8 DPV voltammogram obtained from GNP/GCE for DEX signal in presence of different amount of hydrocortisol in the PBS solutions (pH 7.3).


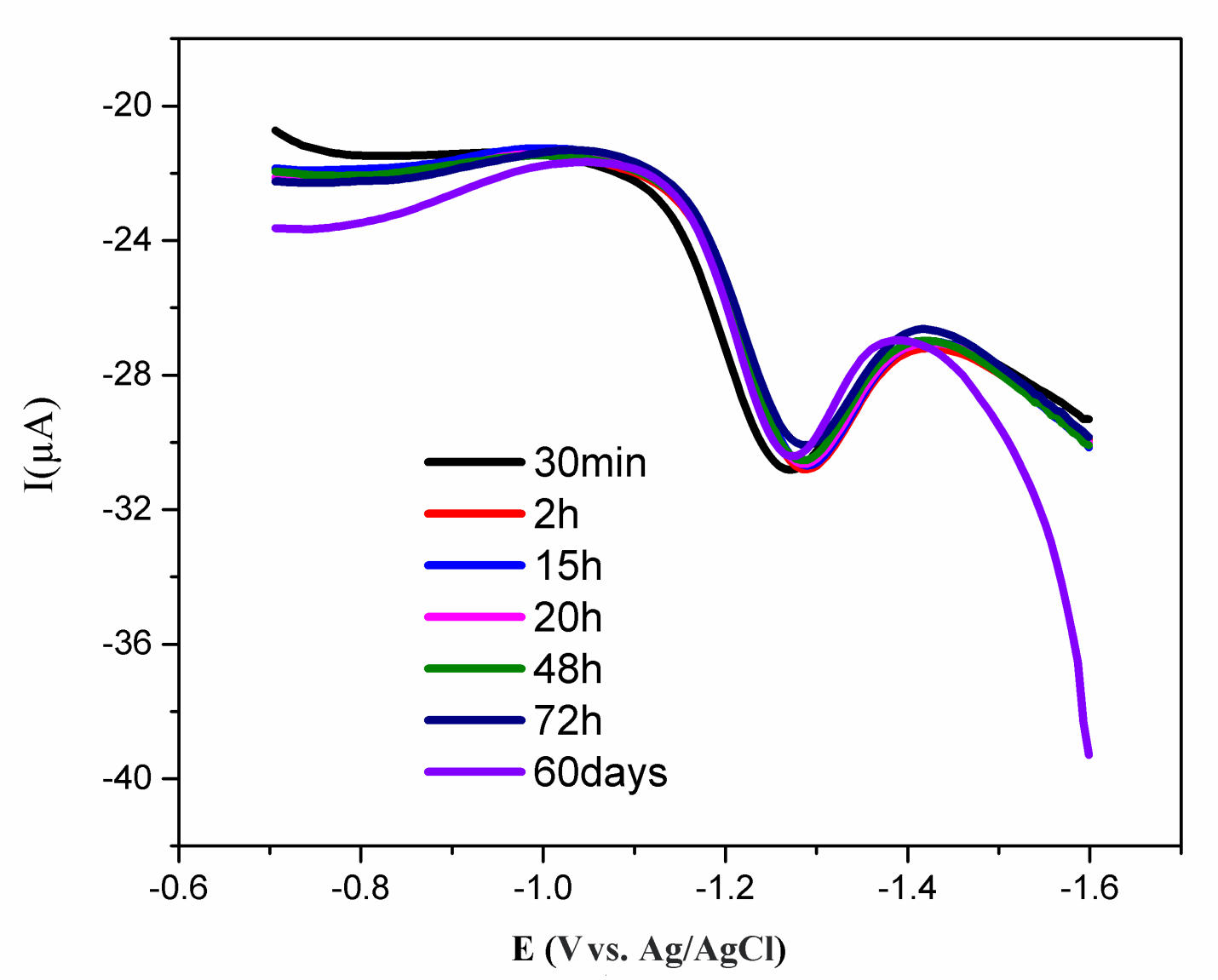


Fig. S9 DPV voltammogram obtained from GNP/GCE in the presence of DEX over the 60 days in the PBS solutions (pH 7.3).


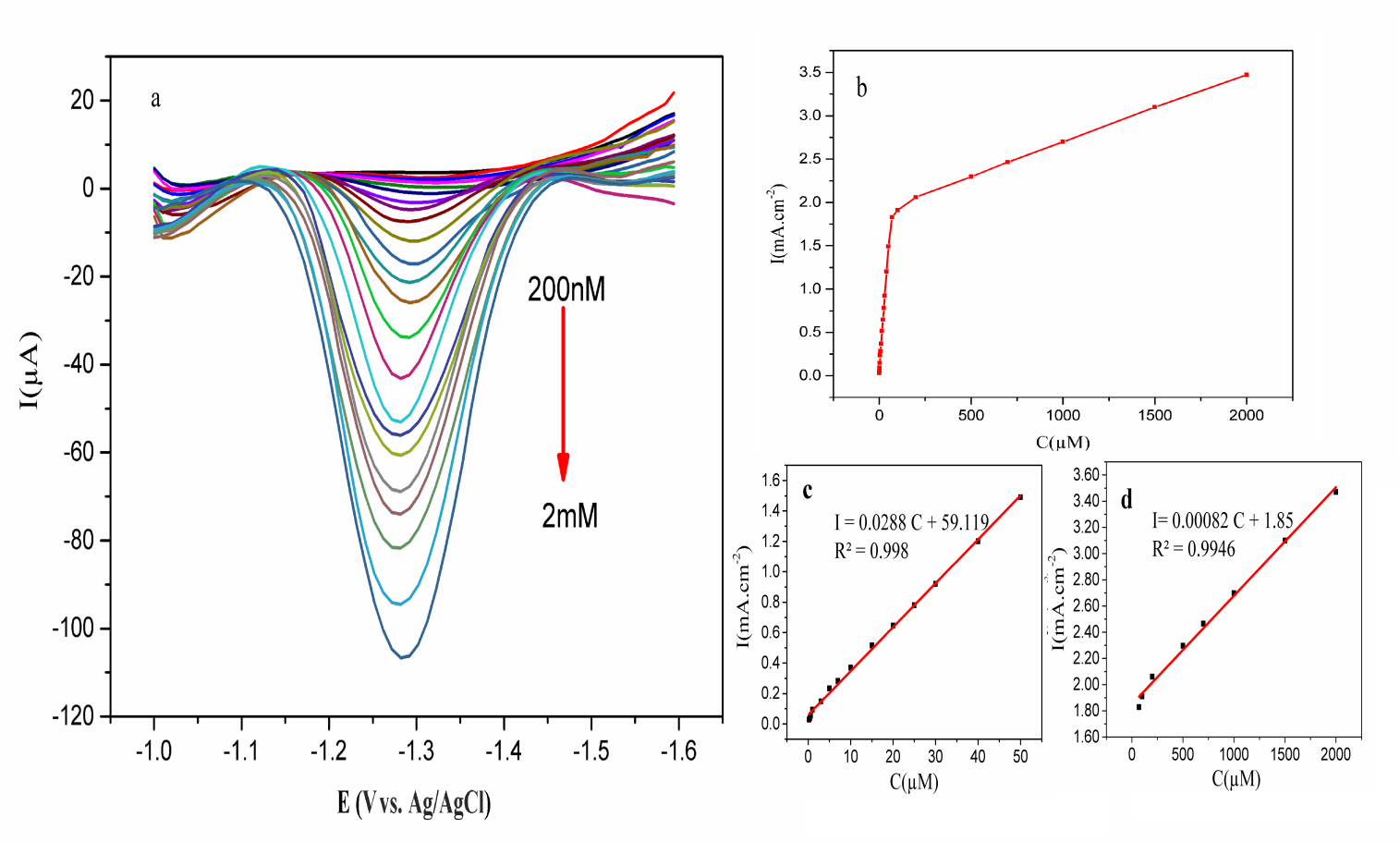


Fig. S10 (a) DPVvoltammogram obtained from the GNP/GCE in the presence of the DEX ranging from 200 nM to 2mM in pH 7.3 human plasma. (b,c,d)calibration plots of the peak current vs. different concentrations of the DEX.
